# Managing COVID-19 within and across health systems: why we need performance intelligence to coordinate a global response

**DOI:** 10.1186/s12961-020-00593-x

**Published:** 2020-07-14

**Authors:** D. Kringos, F. Carinci, E. Barbazza, V. Bos, K. Gilmore, O. Groene, L. Gulácsi, D. Ivankovic, T. Jansen, S. P. Johnsen, S. de Lusignan, J. Mainz, S. Nuti, N. Klazinga, on behalf of the HealthPros Network, P. Baji, O. Brito Fernandes, P. Kara, N. Larrain, B. Meza, A. Murante, M. Pentek, M. Poldrugovac, S. Wang, C. Willmington, Y. Yang

**Affiliations:** 1 Department of Public and Occupational Health, Amsterdam UMC, University of Amsterdam, Amsterdam Public Health research institute, Meibergdreef 9, 1105 AZ Amsterdam, The Netherlands; 2 Department of Statistical Sciences, University of Bologna, Via Belle Arti 41, 40126 Bologna, Italy; 3 Management and Health Laboratory (MeS), Institute of Management and EMbeDS, Scuola Superiore Sant’Anna, piazza Martiri della Libertà, 33 Pisa, Italy; 4 OptiMedis AG, Burchardstraße 17, 20095 Hamburg, Germany; 5 Department of Health Services Research and Policy, London School of Hygiene and Tropical Medicine, Tavistock Place, 15-17 London, United Kingdom; 6 Department of Health Economics, Corvinus University of Budapest, Fővám tér 8, Budapest, 1093 Hungary; 7 Danish Center for Clinical Health Services Research (DACS), Department of Clinical Medicine, Aalborg University and Aalborg University Hospital, Fredrik Bajers Vej 5, 9100 Aalborg, Denmark; 8 Nuffield Department of Primary Care and Health Sciences, University of Oxford, Woodstock Rd, OX2 6GG Oxford, United Kingdom; 9 Psychiatry Management, Aalborg University Hospital, Mølleparkvej 10, 9000 Aalborg, Denmark

**Keywords:** COVID-19, health systems, performance measurement, health services, health information, policy, evidence

## Abstract

**Background:**

The COVID-19 pandemic is a complex global public health crisis presenting clinical, organisational and system-wide challenges. Different research perspectives on health are needed in order to manage and monitor this crisis. Performance intelligence is an approach that emphasises the need for different research perspectives in supporting health systems’ decision-makers to determine policies based on well-informed choices. In this paper, we present the viewpoint of the Innovative Training Network for Healthcare Performance Intelligence Professionals (HealthPros) on how performance intelligence can be used during and after the COVID-19 pandemic.

**Discussion:**

A lack of standardised information, paired with limited discussion and alignment between countries contribute to uncertainty in decision-making in all countries. Consequently, a plethora of different non-data-driven and uncoordinated approaches to address the outbreak are noted worldwide. Comparative health system research is needed to help countries shape their response models in social care, public health, primary care, hospital care and long-term care through the different phases of the pandemic. There is a need in each phase to compare context-specific bundles of measures where the impact on health outcomes can be modelled using targeted data and advanced statistical methods. Performance intelligence can be pursued to compare data, construct indicators and identify optimal strategies. Embracing a system perspective will allow countries to take coordinated strategic decisions while mitigating the risk of system collapse.A framework for the development and implementation of performance intelligence has been outlined by the HealthPros Network and is of pertinence. Health systems need better and more timely data to govern through a pandemic-induced transition period where tensions between care needs, demand and capacity are exceptionally high worldwide. Health systems are challenged to ensure essential levels of healthcare towards all patients, including those who need routine assistance.

**Conclusion:**

Performance intelligence plays an essential role as part of a broader public health strategy in guiding the decisions of health system actors on the implementation of contextualised measures to tackle COVID-19 or any future epidemic as well as their effect on the health system at large. This should be based on commonly agreed-upon standardised data and fit-for-purpose indicators, making optimal use of existing health information infrastructures. The HealthPros Network can make a meaningful contribution.

## Background

In this paper, we make the case that performance intelligence plays an essential role in guiding the decisions of health system actors on the implementation of contextualised Coronavirus disease 2019 (COVID-19) measures and their effect on patients with non-COVID-19 health needs. This should be based on commonly agreed-upon standardised data and fit-for-purpose indicators, making optimal use of existing health information infrastructures.

Performance intelligence can be defined as the structured approach to acting on health policies, using knowledge and information generated by the application of scientific methods to comparable healthcare data to systematically measure indicators of health systems performance. The COVID-19 pandemic is a complex global public health crisis, presenting not just clinical but also organisational and system-wide challenges in the short, intermediate and longer term. In order to manage and monitor this crisis, different research perspectives are needed, including health services research, public health, epidemiology, sociology, behavioural and political sciences, management, and economics. Performance intelligence is an approach that emphasises the need for different research perspectives in supporting health systems’ decision-makers to determine policies based on well-informed choices aiming for a whole system scope and varying time perspectives. Managing the outbreak of COVID-19 thus becomes an integral part of governing healthcare systems and not as a separate track with its own rationale.

The COVID-19 pandemic poses challenges for health systems that go well beyond the current emergency related to the treatment of new cases. A risk from the efforts put into managing the crisis is the crowding out of non-COVID-19 patients, who comprise the vast majority of those in need of care.

To maximise capacity for the care of COVID-19 patients, health systems are deliberately delaying many elective procedures [1–3]. In parallel, it is observed that patients are refraining from accessing care themselves due to their fear of coronavirus contamination in hospital or because they do not wish to burden healthcare workers with their ‘minor’ problems. The effects on patient groups in need of semi acute care are already becoming clear through reported reductions in essential service utilisation in various countries [4, 5]. In addition, there are substantial changes in how non-COVID-19 care is delivered through modified protocols [6]. These changes in the provision of regular care require close monitoring to inform decision-makers about the appropriateness of COVID-19 measures and strategies that can continue to guarantee (the quality of) essential care in this and the next phases of the pandemic.

The capacity of health facilities has rightfully been at the centre of the political debate on the availability of hospital beds and intensive care units (ICU) during the outbreak of COVID-19. In most instances, this has entailed strengthening services with equipment, technology and staffing. On the other hand, the role of primary care has been remarked upon but differently addressed, with related consequences noted over the course of the outbreak. For instance, in the decentralised health system of Italy, the high fatality rate found in the Lombardy region (as opposed to the lower rates in the bordering regions of Veneto and Tuscany) is likely partly explained by differences in the emphasis on acute hospital and primary care and testing capacity [7, 8]. In Veneto, a community-based approach facilitated the testing and isolation of patients [9]. Moreover, in Lombardy, general practitioners, although frequently faced with high-risk situations in caring for their patients at home, were confronted with a lack of personal protective equipment [10, 11] — 14.3% of all COVID-19 cases in Lombardy were among healthcare workers, compared with 4.4% in Veneto, as of 30 March 2020 [9].

Performance measurement, monitoring and reporting can support the balancing of system approaches to respond to COVID-19 — or any other epidemic — with targeted strategies such as accurate predictions of hospital admissions and of primary, home and long-term care needs after discharge. To guide rational and needs-based decision-making, we have embraced the notion of ‘performance intelligence’ as a compelling solution. In this paper, we present the viewpoint of the EU-funded Marie Sklodowska-Curie Innovative Training Network for Healthcare Performance Intelligence Professionals (HealthPros) on how performance intelligence can be used during and after COVID-19 [12, 13]. Our purpose is to present the framework of HealthPros, show how its use can contribute to the resolve of inconsistencies noted in outbreak responses, and show how it can help govern national healthcare services and systems, in synergy with a global research community fully dedicated to the development of such intelligence. As policies and practices need to be compared, while leaving room for different modes of implementation, common knowledge must build up. Performance intelligence is critical to ensuring that each territory, be it a council, district, region or federal state, may choose its own policy based on the best available evidence for a health system response.

### What is performance intelligence and how does it work?

Healthcare performance measurement and its use as performance intelligence plays an important role in guiding the decisions of health system actors [14, 15]. Regional, national and international experiences in this field can be directly translated into solutions that will help manage the COVID-19 pandemic and its longer-term impact on health systems and societies, including during the various transition phases that will gradually allow a return to a new ‘normal’ life.

Comparative health system research, and its transparent and timely reporting, is needed to help countries to shape their response models in social care, public health, primary care, hospital care and long-term care through the different phases of the pandemic from pre-disaster to the reconstruction phases. At each phase in each specific context, there is a need to compare different bundles of measures, whose impact on health outcomes may be modelled using targeted data and the application of advanced statistical techniques. Such research starts with agreeing on the terms for each phase of the COVID-19 pandemic, differentiating countries and geographic areas within countries to specific stages, and contextualising the characterisation by the structure of health systems. Responses will need to address the care continuum of prevention, diagnosis, treatment and follow-up of COVID-19 patients. Each stage will require specific epidemiological approaches that must be capable of considering the regulatory, organisational, political and cultural context.

As defined, performance intelligence can be used to gather comparable data, develop indicators and identify optimal strategies for taking action. For instance, the response to COVID-19 could use performance intelligence to select one or more of the following: (1) considering substitution effects from hospital to primary care or long-term care for specific categories of patients (requiring collaboration between health sectors); (2) swapping tasks between specialities and professions according to capacity needs; (3) undertaking disruptive innovation through novel technologies; (4) substantially improving and optimising the information infrastructure through data linkage of different databases; (5) analysing different possible behavioural models of the population and of health professionals, and the impact of nudging strategies; (6) avoiding disruption in the delivery of the continuum of care.

The rationale on which pandemic response decisions are based will need to be explained. Embracing a system perspective allows countries to take coordinated strategic decisions and ensure essential levels of healthcare for all patients (including those who need routine assistance), while mitigating the risk of system collapse.

**Fig. 1:**
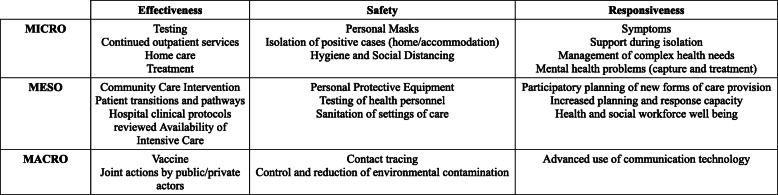
Performance Intelligence Outbreak Preparedness Matrix: dimensions and example of relevant policies

To facilitate a system perspective, performance intelligence offers a policy matrix that can be structured consistent with the Organisation for Economic Cooperation and Development (OECD) framework of health systems performance assessment [14]. According to this model (Fig. 1), three vertical dimensions need to be investigated, namely effectiveness, safety and responsiveness (population needs, access, equity). On the horizontal axis, there are three primary intervention areas at the micro, meso and macro level. We have populated the matrix with some relevant examples that emerged in the debate following the COVID-19 outbreak.

At the micro-level, standardised procedures for COVID-19 patients lack timely examination and are still largely not monitored in a comparable manner. For example, hospitals established different pathways to receive suspected cases, which may eventually lead to the uncontrolled spread of the disease. Similarly, there has been discussion on evidence-based approaches to prioritising groups at high-risk of complications such as patients with specific case-mixes identified from multivariable risk adjustment and on testing for subgroups exposed to the infection or subjects with symptoms. Further, best practices for personal safety remain unclear.

At the meso-level, the role and rules of engagement of professionals and healthcare providers have been quite heterogeneous. Some countries (or regions in decentralised countries) prioritised acute settings, while others invested more in community care intervention and monitoring [16]. The relative merits of different approaches should be formally evaluated in the course of the pandemic to make sure that timely corrective actions may be undertaken to achieve the best possible outcomes.

At the macro-level, there has been enormous pressure on the supply side, even prior to knowing the most effective solutions and, in some cases, even disagreeing on the effectiveness and/or accuracy of some products. All countries are concerned about (potential) shortages of protective personal equipment, tests and ventilators [17]. The demand caused an avoidable competition between countries, later followed by more targeted strategies to convert internal production, with an adequate level of intelligence. Finally, the struggle to increase capacity pushed countries like Ireland and Spain to ‘nationalise’ private facilities in addition to public hospitals [18, 19]. However, it is still unclear whether such solutions offer the highest standards of safety required to assist patients infected with COVID-19.

Performance intelligence is essential to support those decisions through accurate modelling and structured comparison of the systems in place (e.g. ICU), the outcomes achieved (e.g. survival) and the finely tuned population (including non-COVID-19) health needs (e.g. percentage of high-risk susceptible persons). Sharing available expertise within and between countries and capitalising on expertise (e.g. the OECD Health Care Indicators Project and the HealthPros network) may provide a springboard for a global response.

Performance intelligence may help to improve pandemic preparedness in many ways, even for aspects not directly related to the disease itself. A relevant case is that of preventing unwarranted side effects related to the disruption of services for urgent care management (e.g. cancer patients undergoing chemotherapy or people with diabetes under dialysis).

To respond to these challenges, the research activities involved require mobilising a substantial capacity of interdisciplinary experts from the diverse fields of social sciences, public administration, health economics, health services research, political science, health policy and management, biostatistics, clinical epidemiology, medicine, improvement science and health information. Moreover, investing in this capacity through competencies for performance intelligence is essential.

### The role of information infrastructure in enabling performance intelligence

Many countries and regions have adapted their data privacy legislation to increase opportunities for data sharing and are currently relaxing this carefully balanced legislation [20]. This will require continuous close monitoring by organisations such as the OECD and WHO. The response to the disease will require a substantial increased effort to assess how the existing data systems in place, such as clinical registries, administrative data and electronic medical records, next to established infectious diseases surveillance systems can be used for monitoring and surveillance as well as for informing capacity planning and management. Such capacity may be able to assist countries during this and any other unfortunate circumstances that may challenge the resilience of our healthcare systems.

A relevant and still insufficiently addressed issue is monitoring changes over time regarding disparities in quality of healthcare for both vulnerable and at-risk populations. These will require detailed data collection (and linkages) of multiple characteristics such as demographics (age, gender, etc.), proxies of income and education (e.g. socioeconomic status and health literacy), and the clinical and personal case-mix (e.g. comorbidity burden, migration status and ethnicity). This will inform, among others, the required stratification needed to tailor strategies in services delivery, tackling determinants and ensuring access for all.

For any outbreak, multiple databases will have to be linked across sectors to include information from people with and without the disease. That is because risk adjustment should compare multivariable patterns for both exposed and non-exposed groups. For instance, the evaluation of care for COVID-19 patient needs also detailed data on non-COVID-19 individuals, which may not be possible to collect accurately without available population-based data (e.g. using a common ID for a representative control sample of all citizens) [20]. In most cases, this would require implementing routine data collection in regular systems of care (e.g. general practitioner information systems). This would allow sophisticated analysis, for example, to identify selection bias in access to care. Accurate data may also directly allow automated checks of anecdotal reports from clinicians in countries (e.g. Italy) informing patient allocation decisions for the ICU given the scarcity of resources and based on personal preferences and characteristics associated with a higher likelihood of survival (e.g. age and comorbidities) [21, 22]. The British Medical Association has already included this in their latest clinical guidelines [23]. Potential disparities in prevention, diagnosis, treatment and follow-up care should be also monitored. For instance, deprived COVID-19 patients might have a lower likelihood of admission to hospital or ICU, resulting in lower survival. The present crisis should not increase disparities but should rather be seen as an opportunity to capture all relevant data that is necessary to reduce them. Only if we start from a consolidated measurement system already in use, is it possible to measure the impact of the emergency, which is necessary to introduce changes in the actions taken in the event of a problem [15].

An aspect of responsiveness that deserves particular attention during an outbreak such as COVID-19 is that of mental health. The conditions imposed by central governments may lead to elevated levels of anxiety or depression related to the imposed physical distancing, quarantine, lockdown measures or job losses, each of which should be investigated in their own right. This should be closely monitored to address the situation in a timely manner, for instance, by including patient-reported experience and outcome measures (PREMS and PROMS) in regular surveys and integrating this in clinical practice [24]. In most countries, data on the quality of mental healthcare is currently scarce and often limited to utilisation and, at times, to suicide data. Researchers are urged to explore the possibilities to exchange and replicate patient-reported outcome measures/patient-reported experience measures survey questions in their ongoing and future research to contribute to such intelligence. Special attention should be given to assessing the mental health status of healthcare professionals and to interventions to ensure that the healthcare workforce remains healthy [25].

The data needed to populate the proposed matrix may ensure better alignment between needs, demands and actual use of healthcare services, in times of emergency and scarcity of resources. This will require case studies modelled on country experiences and involve considerable investment to scale-up the current information infrastructure. Some countries provide relevant examples. For example, the primary care national surveillance system of the Royal College of General Practitioners Research and Surveillance Centre in England has a mandate to monitor the occurrence of cases for COVID-19 [26]. Also, countries like Finland (Findata) and South Korea (HIRA) demonstrate how their present information infrastructure can be put at the service of the nation to manage this crisis [27]. These cases may help in identifying opportunities, forming recommendations and sharing lessons across countries on how to optimise the existing information infrastructure for performance intelligence in a coherent and standardised manner [28].

### How to implement a performance intelligence framework to inform policies against COVID-19

The heterogeneous responses of governments threatened by COVID-19 did not seem to prioritise the need for creating a common performance intelligence framework as a basis for common evidence-based policies [29]. Experts have struggled to deliver stable forecasts, using models that rely on aggregate and partly inaccurate data, contributing to alarming estimates of death that required later adjustments [29–31]. During the first 3 months of the pandemic, the usual dissemination means, including research abstracts and publications, were used to inform governments, not infrequently with contrasting messages. A common problem of this approach is that the data underpinning results such as comparisons between fatality rates and projected trends of pandemics are mostly aggregated and not fit for purpose to contribute to specific models.

**Fig. 2:**
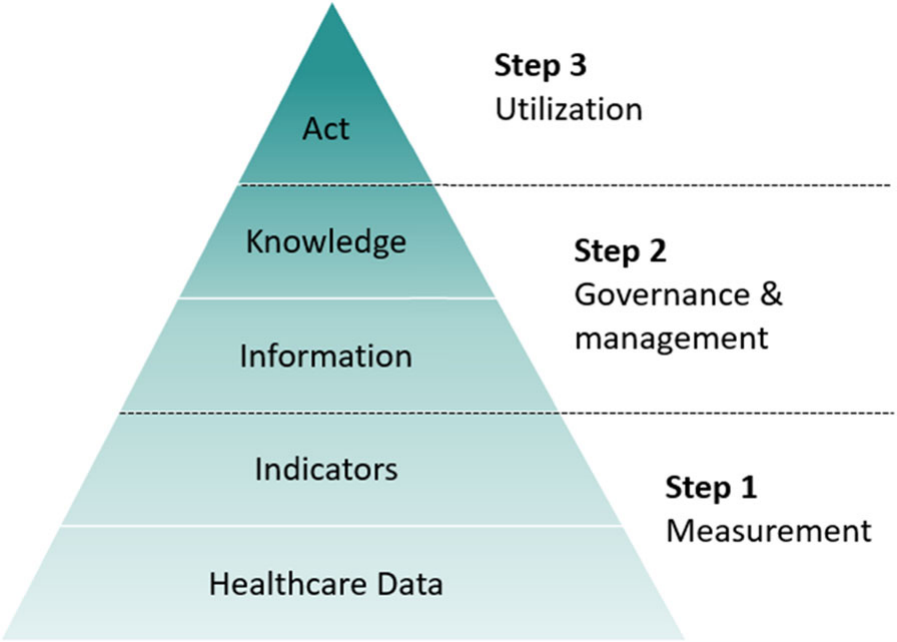
The hierarchical structure of a performance intelligence framework

A framework for the implementation of performance intelligence has been outlined by partners of the HealthPros Network [12, 13]. The diagram (Fig. 2) presents a hierarchical structure with healthcare data and indicators at the base of performance measurement. These are fundamental elements that have been found to be weak since the inception of COVID-19, notwithstanding the recent expansion of electronic information systems that accelerated the speed, increased the volume and extended the range in types of data available [32].

During the current COVID-19 crisis, we have observed the paramount importance of available data with the right information, in the right hands at the right time. Although different platforms have evolved overnight [33, 34], the challenge is not a shortage of the technology or our ability to collect data but rather their meaningfulness. Developing our understanding of what makes an indicator actionable for decision-makers is of critical importance to ensure that we fully leverage our data in day-to-day operations and in times of health emergencies. Such leverage will require the standardisation of definitions, recording, use of ontologies and common data models to enable international comparison. First steps have been created on Bioportal [35, 36]. In times of crisis, trust in data should not be overlooked. While in public announcements WHO and the European Centre for Disease Prevention and Control did not compare the number of confirmed cases and case fatality of COVID-19, country comparisons are made in their dashboards [37, 38]. These hide differentiation in testing policies and in definitions of mortality and case calculations (case ascertainment), include no mentioning of mean age or comorbidities, and are severely affected by a disproportionate lack of coding COVID-19-related deaths in nursing homes; these features leave room for misinterpretation [39]. Such global rankings are therefore of limited utility.

At a higher level in the pyramid of performance intelligence we find the need to translate indicators into the knowledge and information used for governance. At this level, decision-makers have a specific purpose to use healthcare performance data in relation to the COVID-19 pandemic across the macro, meso and micro-levels of the health system outlined above. Therefore, at the highest level of the pyramid, we position the need to utilise data and knowledge in order to act in response to a specific problem. Each purpose demands different information for decision-makers. Particularly in times of emergency, different methodological considerations will determine the actionability of performance intelligence. The time-sensitive nature of indicators and the need for standardised data are critical factors. Standardisation enables the comparability and analysis of indicators and facilitates trust in data.

At the two opposing ends of the pyramid we position management and dissemination. Implementation of the framework within and across countries may be particularly complex, requiring coordination and facing many barriers (such as data protection rules and data exchange) that can be hampered by administrative hurdles (e.g. decentralisation) and international boundaries. Dissemination is key to making information accessible to the broader population. The general public uses data on COVID-19 to form an opinion on the success of their governmental response and determines trust in their responsiveness. While the underlying methodology cannot be simplistic, the availability of easily interpreted data is particularly important. The public is overwhelmed by the availability of popular indicators such as daily case fatality and incidence rates. Evidently, most measures hide time lag effects (e.g. test results relative to samples taken days before and/or hospitals reporting data asynchronously) or may be far from accurate (e.g. under-estimated mortality rates due to denominators biased by limited testing capacity). As a result, the magnitude of indicators published in the public domain often does not match public statements from policy-makers about the effect of their policies. For example, a policy-maker in charge of an ICU may plan capacity 2 weeks ahead based on today’s infection incidence rate. If that rate decreases, the plan will be optimistic and not aligned with expectations of the public.

Good quality data is pivotal for intelligence during the COVID-19 pandemic and beyond. In HealthPros, we invest in training a future workforce with performance intelligence competencies, which directly links the creation of a new generation of performance intelligence professionals to all layers of pyramid of intelligence. This is much needed to ensure preparedness for the transition strategy of COVID-19 and any possible other unfortunate circumstances that may challenge the resilience of our healthcare systems for which countries can find this framework ready for use.

Through the Innovative Training Network, we have access to many different databases and hold interdisciplinary expertise. Here, we see the opportunity but also feel the obligation to apply the project to address a number of questions that are key to assuring the proper functioning of health services and use of health data during epidemics.

## Conclusion

Performance intelligence plays an essential role as part of a broader public health strategy in guiding the decisions of health system actors on the implementation of contextualised measures to tackle COVID-19 or any future epidemic and their effect on the health system at large. This should be based on commonly agreed-upon standardised data and related systems of indicators, making optimal use of existing health information infrastructures. We urge policy-makers to support the development and use of performance intelligence based on this vision and invite other health services researchers to join and further strengthen this approach for the enduring transformation of pandemic-resilient health systems.

## Data Availability

Not applicable.

## Abbreviations

COVID-19: Coronavirus disease 2019
HealthPros: Healthcare Performance Intelligence Professionals
ICU: Intensive care unit
OECD: Organisation for Economic Cooperation and Development
